# Summary of the Pediatric Pain Management Symposium 2018 in Boston, MA

**DOI:** 10.1097/PR9.0000000000000683

**Published:** 2018-09-11

**Authors:** Christine Greco

**Affiliations:** *Boston Children's Hospital, Harvard Medical School, Boston, MA, USA*

The IASP Satellite Symposia on Pediatric Pain Management: State of the Art and Science will be held from September 10 to 11, 2018 in Boston, MA, just prior to the official start date of the IASP World Congress on Pain. The conference will include internationally known speakers and will focus on several prominent themes including pediatric chronic pain and rehabilitation, pain in children with developmental disabilities, opioid use and misuse, pain and fear related to procedural pain in children, and pain as a consequence of critical illness. The sessions will integrate basic, translational and clinical science with clinical practice through a mix of lectures, audience discussions, and roundtable presentations. The conference will bring together participants from multiple disciplines to share ideas and new developments in the care of children with pain.

Sessions focused on pain as a consequence of critical illness on day 1 will begin with a talk by Laura Cornelissen, PhD on the impact of prolonged anesthesia and sedation in infancy and on early childhood development. Suellen Walker, PhD will discuss the impact on neurobiology of early life surgical trauma in animal models. Martha Curley, RN, PhD will review outcomes of clinical trials of opioid and sedation regimens for mechanically ventilated children and how it relates to weaning practices in children. Shifting the focus to chronic pain and rehabilitation, Laura Simons, PhD will present on the relationship of fear and brain circuitry in the context of interdisciplinary pain treatment. Tanja Hechler, PhD will present research findings on the effect of fear response on the interpretation of internal body signals in adolescents with chronic pain and how psychological interventions can be directed to reduce the fear response.

Afternoon sessions on day 1 continue on themes related to rehabilitation and chronic pain. Michael Sangster, PT will discuss the application of novel brain based physical therapy interventions for the management of chronic pain in children and how the interventions can be used to induce neuroplasticity. The following talk will be given by Richard Wicksell, PhD who will present the use of Acceptance and Commitment Therapy (ACT) to minimize the influence of pain by increasing the ability to have effective behavioral patterns. The next presentation by Charles Berde, MD, PhD, Boris Zernikow, MD, PhD, and Stefan Friedrichsdorf, MD will be a roundtable discussion on how the variable trajectories of life limiting illnesses can shift treatment to include aspects of palliative care with rehabilitative treatment. Maria Fitzgerald, PhD will finish day 1 with a session on translational pain research in infants, specifically in understanding mechanisms of neuroplasticity and the long-terms effects of pain on the developing brain. Sessions on day 2 will begin with a talk by Joe Kossowsky, PhD on clinical outcome research in pediatric pain in the era of digital health and precision medicine. Shannon Manzi, PharmD will present the application of pharmacogenomic testing and how it can be used in algorithms for pain management. Alyssa LeBel, MD and Monique Ribeiro, MD will discuss the clinical diagnosis of Somatic Symptom and Related Disorders and treatment options to address a child's functional disability and distress. Data on the use of functional neuroimaging in these disorders will also be presented. Following this will be a talk by Neil Schechter, MD, Heather Molind, DPT and Anne Louise Oaklander, MD on complex and sometimes puzzling pain conditions in children that are often disabling, such as Amplified Pain, Ehlers Danlos syndrome, Primary Pain Disorder, and Small Fiber Neuropathy. They will co-present dilemmas in diagnosing, treating and conceptualizing these painful conditions.

For the remainder of the morning, the focus will shift to topics related to pain in children with developmental disabilities. Julie Hauer, MD will present the challenges of identifying pain sources in children with severe impairment of the central nervous system and effective pharmacologic and nonpharmacologic management of pain. Brian Snyder, MD will review surgical implications for children with neuromuscular diseases and the management of postoperative concerns such as surgical pain and spasticity. The afternoon sessions on day 2 will focus on various themes related to opioids and the opioid crisis. Sushma Bhatnagar, MD will begin with a presentation on barriers to pain relief in children, particularly in developing countries, such as non-availability of opioids and misconceptions about opioids and pain management in children. Sharon Levy, MD, Christine Greco, MD, and Benjamin Lee, MD will co-present on the safe and careful use of opioids in primary care, chronic pain clinics and in-hospital settings and how the opioid crisis has led to changes in opioid use and practice in these settings. Judy Ashworth, MD will discuss innovations in developing analgesics that are equally effective as currently available opioids but with less abuse and addiction potential. Later in the afternoon, Alana Arnold, PharmD and Stefan Friedrichsdorf, MD will present consequences of untreated and recurrent needle pain and the use of topical local anesthetics and nonpharmacologic strategies to treat pain from needle procedures. A talk by Susan Graca, MS will follow on clinical interventions to reduce pain and fear in children undergoing medical procedures, particularly as they relate to different developmental stages. The final presentation of the conference will be given by Melanie Noel, PhD who will present data on how children's memories of pain can influence future pain experiences and the impact parents can have to modify children's memory of pain.

The following are summaries of sessions and abstracts of poster presentations focusing on the prominent themes of the conference.

## Approaches to needle pain procedures

Alana Arnold, PharmD, BCPS, AQID

Boston Children's Hospital, Harvard Medical School, Boston, USA

Needle stick is associated with pain, anxiety and distress in pediatric patients and their families. There is evidence that long-term consequence of this potentially avoidable pain may shape a patient's response to future painful events. Negative consequences associated with repetitive needle pain procedures can be reduced and, in some cases, prevented with a multimodal pain management approach through both non-pharmacologic and pharmacologic interventions.

Non-pharmacologic interventions performed by child life professionals, such as distraction and relaxation techniques can help decrease anxiety and fear and can be used with pharmacologic agents as a comprehensive approach to needle pain procedures.

There are many pharmacologic options for the prevention of needle pain in children. The selection of agent depends on age of patient, ease of use, urgency of procedure, onset of action, effectiveness, side effects and cost-effectiveness. The administration of local anesthetic is often chosen as a first-line agent due to ease of application, reliability, and safety of side effect profile.

All needle pain prevention options will be reviewed with an emphasis on aspects of advantages and disadvantages and clinical application of the various modalities.

## The quest for less: in search for potent analgesics with less abuse potential

Judy Ashworth, MD

Gruenenthal GmbH, Germany

With the US in the midst of an Opioid Crisis, the need for strong analgesics which are as effective as classical opioids but with less potential for abuse, diversion, overdose and addiction has never been higher. At the same time, the scientific and regulatory challenges coupled with difficulties with formulary coverage post-approval have led to what appears to be an exodus of mid-sized and big pharma from this space.

In her talk, Dr Ashworth addressed the questions (1) How has industry been responding to the crisis in terms of scientific innovation? and (2) What innovations are on the horizon which could potentially help address the crisis of prescription opioid abuse while at the same time not leave the needs and concerns of pain patients unattended? Her overview included a review of abuse deterrent formulations, biased opioid agonists and cannabinoids along with novel non-CNS acting targets (eg, Nav1.7, Ca2.2, TRPV1, anti-NGF), improved local anesthetics and some non-pharmacological approaches.

## Impact of prolonged anesthesia, sedation and analgesia in infancy: insights into early childhood development

Laura Cornelissen, PhD

Boston Children's Hospital, Harvard Medical School, Boston, USA

Millions of children are exposed to general anesthesia for elective surgery each year. Commonly used anesthetics powerfully modulate important processes to prevent pain and awareness, as well as processes that are also involved in the fine-tuning of brain development.

Clinicians are concerned about the long-term outcomes of exposure to anesthesia when given at high doses, prolonged duration or with repeated exposures in early-life ie, impaired cognitive and behaviour traits. Accordingly, the US FDA has recently changed labelling of anesthetic agents warning against repeated or prolonged exposure in young children.

Given that prospective clinical outcome studies are time-consuming, expensive to conduct and run risk of low attrition rates, more information is needed on the immediate effects of drug exposure on brain health to guide clinical practice. Recent work using non-invasive brain monitoring approaches, such as electroencephalography provide important information about brain-health.

Dr Cornelissen will present ongoing research examining the early childhood development in the context of prolonged anesthesia, sedation and analgesia. She will show data examining perioperative brain dynamics in cohorts of infants with exposure to general anesthesia and sedation and their association with specific developmental trajectories.

## Clinical trials and outcomes of analgesia and sedation for mechanical ventilation

Martha Curley, PhD, RN, FAAN

Children's Hospital of Philadelphia, University of Pennsylvania, Philadelphia, USA

Ensuring the safety and comfort of critically-ill children is integral to the practice of pediatric critical care. Humane care includes creating a healing PICU environment and using medications to achieve the therapeutic goals of analgesia and anxiolysis while facilitating life-sustaining therapies. From days to weeks, critically-ill children frequently receive various combinations of opioids and sedatives. Although there are compelling short-term benefits in using sedation in children who are unable to cognitively understand the imperative nature of critical care instrumentation, we now know that sedative use is associated with iatrogenic injury. Specifically, sedation contributes to immobility-related complications. Over time, drug tolerance develops and may precipitate iatrogenic withdrawal syndrome when the sedative agents are no longer necessary. In addition, there is increasing concern that commonly used PICU sedatives may harm the developing brain and impact long-term neurocognitive outcomes.

Significant improvements have been made in the comfort management of critically ill children. For example, we now have valid and reliable assessment instruments to adequately describe the multifaced construct of comfort in critically ill children. These instruments have facilitated the use of targeted therapies with the goal of PICU sedation evolving from an unresponsive critically-ill child to one who is calm, easily aroused, and readily evaluated. In the *RESTORE* clinical trial we were able to demonstrate that among children undergoing mechanical ventilation for acute respiratory failure, the use of a PICU nurse-implemented, goal-directed sedation protocol, compared with usual care, did not reduce the duration of mechanical ventilation but was associated with a different sedation experience. Specifically, children managed on the *RESTORE* protocol were safely managed in a more awake and calm state while intubated; received fewer days of opioid exposure and sedative classes; and did not experience an increase in inadequate pain or sedation management or clinically significant iatrogenic withdrawal.

Beyond the PICU, clinicians are now focused on the long-term impact of critical illness. We are in the process of completing *RESTORE-cognition*, a follow-up study designed to explore the long-term effects of sedative medications, as currently used in the PICU, on neurocognitive outcomes of infants and young children. In addition to informing the work on Post Intensive Care Syndrome in children (PICS-p), this line of inquiry has implications for the current practice of pediatric critical care; specially, which sedatives are associated with the least long-term iatrogenic injury, thus improving the health and quality of life of children and their families after pediatric critical illness.

**Acknowledgements:** These studies were supported by National Heart, Lung, and Blood Institute (U01 HL086622 and U01 HL086649) and the Eunice Kennedy Shriver National Institute of Child Health & Human Development (R01 HD074757).

## Translational pain research in infants: future directions

Maria Fitzgerald, FMedSci, FRCA, FRS

University College London, London, United Kingdom

While great strides have been made in the measurement and treatment of pain in infants, it is now clear that analysing pain on the basis of stereotypical behaviors and physiological reactivity alone, is insufficient. Thus, treatments that blunt infant pain behaviour may be of limited benefit in reducing pain activity in the infant brain. Brain activity is of critical clinical importance as it can trigger prolonged pain states and cause long term changes in brain development.

Research in animal models has shown that, while the brain cortical structures and connections required for body sensation form early, key functions such as discrimination of a noxious event and localization on the body surface emerge over the first postnatal weeks. There is evidence that the maturation of brain pathways involved in emotional aspects of pain, such as the amygdala, mature later than sensory aspects of pain, such as the somatosensory cortex. This is important for understanding how to treat pain in early life. Descending systems, arising from brainstem structures, which are especially important for contextual control of pain experience, undergo great postnatal change, which impacts on the ability, or otherwise, of infants and children to recruit endogenous pain control. The postnatal maturation of cortical nociceptive and descending pathways makes them vulnerable to experience dependent plasticity and this could explain the long-term effects of early life pain upon the developing brain. Laboratory research into the biological mechanisms underlying this plasticity will help us to prevent adverse consequences.

## Practical aspects of working with children with pain and fear undergoing medical procedures

Suzanne Graca, MS, CCLS

Boston Children's Hospital, Harvard Medical School, Boston, USA

Children may be exposed to a wide range of potentially painful experiences while in the hospital environment, many of which may be difficult for a young child to fully comprehend. A fundamental aspect in caring for hospitalized children undergoing medical procedures is expertise in how children of varying developmental stages understand and communicate about pain and fear. Our group of child life specialists works collaboratively with the multidisciplinary healthcare team to provide support to hospitalized patients and their families. A number of developmentally appropriate nonpharmacologic techniques and strategies can be used to improve coping ability and to minimize a child's pain during painful procedures. Many pharmacologic strategies can complement pharmacologic strategies such as topical local anesthetics in formulating a comprehensive pain management plan. In this lecture, I will discuss evidence for the use of techniques such as comfort measures, guided imagery, breathing techniques, play therapy, and utilization of technology for pain management for procedural pain as well as for acute and chronic pain in other settings.

## Use of opioids in hospitalized children

Christine Greco, MD

Boston Children's Hospital, Harvard Medical School, Boston, USA

Widespread opioid misuse has led many to re-evaluate our approach to the management of pain related to surgery and medical illnesses in hospitalized children. Considerations for the management of acute postoperative pain involves multiple aspects of perioperative care including thoughtful preoperative planning to assess risk of opioid misuse and to ensure that patients and parents have realistic expectations about postoperative pain management. Some patients with anxiety and high fear of pain related to their surgery may benefit from brief psychological counseling in preparation for their surgery. A preoperative referral to a multidisciplinary pain program should be considered for select patients who experience chronic pain or who have had difficult to control pain after prior surgical procedures. Analgesic regimens should include non-opioid analgesics and regional techniques designed to provide optimal, opioid-sparing analgesia. The management of pain in children hospitalized with medical illnesses such as Sickle Cell disease, pancreatitis, and rheumatologic disorders often includes opioids, however algorithms that include non-opioid analgesics and the select use of regional blocks may provide good pain relief and help to reduce overall opioid requirements. Prior to discharge, patients and parents should receive education about safe opioid practices, including how to safe guard medications at home to avoid the risk of inadvertent exposure in younger children. Prescription practices should reflect amounts of opioid needed based on the intensity and duration of pain severe enough to require opioids. Hospital-wide communication across providers and documentation in the electronic record can help to ensure coordination of opioid prescribing. Patients who have persistent pain at post-discharge follow-up, particularly those who continue to require opioid treatment should be evaluated at a chronic pain program.

## Intractable pain and irritability in children with severe impairment of the central nervous system

Julie Hauer, MD

Boston Children's Hospital, Harvard Medical School, Boston, USA

Irritability indicates an abnormal response to stimuli or physiological arousal that can be in response to pain, medications, an emotional state, an acute illness or medical condition. This session will focus on pain in children with severe impairment of the central nervous system (CNS), a frequent and significant problem occurring daily to weekly with high pain severity in those with the greatest impairment. Challenges include uncertainty in identifying pain in nonverbal individuals. Availability heuristics can keep the focus on commonly considered problems, including spasticity and gastroesophageal reflux disease, and interfere with consideration of chronic pain sources such as central neuropathic pain. In those with recurrent pain episodes, there can be more than one source to manage. This session will provide a framework to these challenges. This will include (1) a distinction between pain sources with tests, such as urinary tract infection, in contrast to sources without tests, such as central neuropathic pain; (2) consideration of intermittent muscle spasms and hyperkinetic movement as both primary problems due to the impaired CNS and features observed with any pain source; (3) management of recurrent pain episodes with medications and non-pharmacologic strategies; and (4) the difference between acute pain compared to acute on chronic pain. In children with effective medication management of recurrent pain episodes, discussion will include care plans that manage breakthrough symptoms, balanced with considering when to assess for a new nociceptive source and when to add another medication with a different mechanism of action.

## Transforming clinical outcome research in pediatric pain in the era of digital health and precision medicine

Joe Kossowsky, PhD

Boston Children's Hospital, Harvard Medical School, Boston, USA

Digital and Genomic revolutions have given rise to a new era of individualized medicine where novel biomedical discoveries are leading to more effective prevention, treatment, and diagnosis of disease. While other fields of medicine are starting to harness the potential of personalized medicine, the understanding of how chronic pain conditions develop, progress and are managed remains a challenging task, especially in pediatrics.

Evaluation of progression of chronic pain conditions, including the benefits of interventions is typically done with the information provided by the patients at the moment of the medical encounter. Although valuable information can be collected during clinical visits, this information usually reflects a “snapshot” of the patient condition. In this talk, various phenotyping methods will be summarized that complement conventional clinical characterization of pain and functioning in our pediatric patients. These allow us to quantify relevant behavior both during and between study visits using multi-modal data acquisition in combination with iterative computational approaches. Analytical methods will be introduced that assess the selective genetic associations with patient subtypes defined on the basis of individual symptoms and behavioral and digital phenotypes. Combining these digital outcomes with clinical data from the electronic health records in order to track patient's symptoms, behaviors, functionality and quality of life over time in response to interventions are key components in the development of more effective and personalized pain treatment.

## Somatic symptom disorders: sense and sensibility

Alyssa LeBel, MD, Monique Ribeiro, MD

Boston Children's Hospital, Harvard Medical School, Boston, USA

Somatic Symptom and Related Disorders (SSD), previously known as Somatoform Disorders, is a diagnostic category that describes the presence of distressing somatic symptoms and abnormal thoughts, feelings and behaviors with associated health concerns and functional impairment. While some subcategories, such as Conversion Disorder, are defined by evidence of incompatibility between the symptom and recognized medical conditions, the lack of a medical explanation is neither necessary nor sufficient to make an SSD diagnosis, which is frequent comorbid with both medical and psychiatric diagnoses.

Individuals with these conditions primarily present in medical rather than mental health settings, but tertiary referral for confirmation may advance acceptance of appropriate treatment and limit unnecessary laboratory evaluation. A comprehensive medical and psychiatric evaluation is ideal. With empathy and education, a therapeutic alliance may be initiated, a structured plan of regular follow-up care may be accepted, and an adequate validation of physical and emotional suffering may be possible.

While clinical interview and examination are paramount, the use of standardized inventories can help objectify the assessment. The Children's Somatization and the Functional Disability Inventory, address the child's functional disability, school absenteeism, and somatic symptom reporting.

Although the use of functional neuroimaging (fMRI) of the brain in patients with somatic symptom disorders is nascent, especially regarding pediatric patients, it is a useful and often persuasive tool for patient and family education. fMRI has also provided data regarding changes in brain activation, cortical thickness, metabolism, and circuitry elucidating features of the complexity of the positive (dystonia; pain; non-epileptiform seizures) and negative (paralysis; blindness; numbness) neurologic features and the excessive thoughts, feelings, and anxiety reported in this disorder. Using the visual aids of fMRI and PET scans may increase patient and family acceptance of the neurorehabilitative approach required for functional recovery.

In this lecture, we will review evidence based psychological, psychiatric, and medical approaches to SSDs, particularly when pain is a frequent complaint, and emphasize the importance of a multi-modal treatment plan for these patients and families.

## Opioid stewardship and comprehensive pediatric pain management in a children's hospital health system

Benjamin H. Lee, MD, MPH

Texas Children's Hospital, Houston, Baylor College of Medicine, USA

The increasing number of opioid-related deaths has focused attention on the use of opioid medications in children and adolescents. As a result, many hospitals and health systems are focusing on the role of opioid stewardship and the broader context of comprehensive pain management for patients. In this lecture, the following items will be discussed: the implementation of organizational culture and policies to promote comprehensive pain management, the rationale for consideration of an opioid stewardship program, the development of a systems-wide program with an emphasis on the process of pain management, and the use of clinical systems integration and structure for the measurement of clinical outcomes. The National Quality Forum (NQF)'s model for opioid stewardship will be discussed as a potential framework for developing and implementing an opioid stewardship program; this program centers on 7 fundamentals: leadership, commitment and culture; organizational policies; clinical knowledge and expertise; patient and family caregiver education and engagement; tracking, monitoring, and reporting; accountability; and community collaboration. The development and structure of the model at Texas Children's Hospital will be discussed including barriers, resources needed, and lessons learned from the ongoing implementation of our program.

## Managing the opioid epidemic in the primary care setting

Sharon Levy, MD

Boston Children's Hospital, Harvard Medical School, Boston, USA

The US has declared the opioid epidemic as a public health emergency, and adolescents and young adults are at the epicenter. Medication treatment (MT) for Opioid Use Disorder (OUD) is the clear standard of care for adults, and the American Academy of Pediatrics recommends MT for youth with OUD because it effectively reduces opioid use. Currently, fewer than 25% of youth identified with OUD are prescribed medications; for those younger than 18, as few as 1% receive MT. These low rates of MT for youth are driven in part by a dearth of providers trained and waivered to manage OUD among youth, and in part by an under-developed infrastructure for case-identification and referral. Very few substance use disorder (SUD) specialty programs provide youth-specific programming and overall, youth who do enter treatment fare worse in treatment compared to adults. The stigma and burden of accessing care at a specialty SUD provider presents a substantial barrier for many youth. Integration of treatment for OUD into pediatric primary care presents an opportunity to benefit from fundamental core competencies in developmentally-informed care for this population. Increased competency in managing OUD may also assist primary care offices in developing robust primary and secondary prevention strategies for adolescents. This presentation will review the challenges and opportunities for OUD treatment for youth in primary care.

## Variants in pain management

Shannon Manzi, PharmD, BCPPS, FPPAG

Boston Children's Hospital, Harvard Medical School, Boston, USA

The practical clinical application of pharmacogenomic (PGx) testing in pain management is an evolving skill that will be required as we move further into the era of personalized medicine. Patients are undergoing testing and receiving results from healthcare institutions as well as direct-to-consumer labs and expect their healthcare provider to be facile in interpreting and applying those results. An understanding of the basic tenants of PGx testing modalities, critical interpretation of the results, and incorporation into decision support algorithms in the electronic health record will be discussed in the context of pain management scenarios. Available tools for the primary care prescriber, specialist and pharmacist will be reviewed. Dr Manzi will also highlight some of the current research questions in the field.

## Remembering the pain of childhood: the development, impact, and modification of children's pain memories

Melanie Noel, PhD, RPsych

Alberta Children's Hospital, University of Calgary, Alberta, Canada

Within hours after birth, infants form memories of pain that influence their pain and distress at subsequent painful procedures. Children who develop negatively distorted memories of painful medical procedures are more likely to experience more pain and distress at future procedures. Memories for pain are a more important predictor of subsequent pain than the initial pain experience. Although children's memories of procedural pain can be modified and positively reframed to improve future pain experiences, the development of children's pain memories remains poorly understood, as does how memory can be targeted by parent-led interventions. Dr Noel will present new longitudinal data in children and adolescents undergoing surgery that reveals the influence of anxiety on pain memory development. Her data shows that parents do not reminisce with young children in adaptive ways about past events involving pain, suggesting that parents are not socializing children optimally about pain. Moreover, the ways in which parents talk to their children about past painful procedures can lead to negatively distorted pain memories, placing children at risk for adverse pain and health outcomes. Certain reminiscing styles and content foster more accurate/positive pain memories. Dr Noel will also describe the development of the first parent-led pain memory intervention that is currently being tested in an RCT in the context of surgery and vaccine injections. Given that children's pain memories are one of the most robust predictors of future pain, this work is critical to revealing ways to modify parent-child interactions in interventions to foster more adaptive pain outcomes.

## Controversial diagnoses and labels: amplified musculoskeletal pain, primary pain disorder, Ehlers-Danlos, small-fiber neuropathy

### The value of rehabilitation

Heather Molind, PT, DPT, PCS, ATC

Boston Children's Hospital, Harvard Medical School, Boston, USA

As the identification and diagnosis of pediatric chronic pain conditions becomes more prevalent, physicians continue to identify an increase in the variability of symptoms. This is typically associated with an increasing amount of physical disability and limited engagement in age-appropriate activities. The role of physical therapy and functional rehabilitation is becoming increasing supported in the literature and therefore should be included as a primary treatment modality for pediatric chronic pain management. Additionally, other alternative therapies have been suggested to improve treatment outcomes. In conjunction with presentations from Dr Neil Schechter and Dr Anne Louise Oaklander, this presentation is designed to review the variety of pediatric pain diagnoses that are becoming more prevalent in physical therapy clinical practice, and to identify use of functional rehabilitation as primary modality for treatment.

### Early onset small-fiber polyneuropathy–a treatable cause of “idiopathic” widespread chronic pain in youth

Anne Louise Oaklander, MD, PhD

Massachusetts General Hospital, Harvard Medical School, Boston, USA

Peripheral polyneuropathy, although affecting ∼3.5% of the population, was considered rare in children. Small-fiber polyneuropathy (SFPN) causes widespread chronic pain and dysautonomia, including POTS, abdominal pain/dysmotility, chronic fatigue, and often deconditioning, depression, and disability. We and others have reported evidence of SFPN in ∼40% of adults with “fibromyalgia” (Oaklander et al., *PAIN* 2013), so it is common. We also objectively diagnosed SFPN in 59% to 76% among 41 youngsters with unexplained widespread chronic pain (Oaklander, Klein, *Pediatrics*, 2013). This early onset type seemed caused by autoimmunity involving autoantibodies.

Unlike fibromyalgia, SFPN diagnoses can be test-confirmed, and medical causes often corrected. Lower-leg skin biopsy with measurement of epidermal neurite densities is the gold-standard test. Mass General—the only lab with pediatric norms—has evaluated ∼400 children. The few with genetic SFPN require disease-targeted treatment. NaV mutations require sodium-channel blockers, eg, carbamazepine, mexiletine. Children with HSAN-1 mutations should be considered for l-serine, newly trial-tested in adults (Fridman et al., *Neurology*, 2018).

Most eoSFPN appears autoimmune, with acute illnesses like Guillain-Barré syndrome (Paticoff et al., Anesthesia Analgesia, 2007), and longer ones resembling chronic inflammatory demyelinating polyneuropathy (CIDP). Disabled children are managed with first-line immunotherapies for immune neuropathy, corticosteroids and intravenous immunoglobulin (IVIg). Our 55-case series of apparently autoimmune SFPN patients treated with IVIg (Liu et al., Ther Adv Neuro Disorders, 2018), included children as young as 6 years. Both pain scores (*P* = 0.007) and objective biomarkers (autonomic testing; *P* ≤ 0.001) improved. Seventy-four percent rated themselves “improved” and neurologists labeled 77% as “IVIg responders”; 16% entered remissions sustained after IVIG withdrawal.

Thus, diagnosing SFPN in children with unexplained widespread pain permits more-effective, disease-modifying treatments.

### Controversies in the labelling and diagnosis of non-specific disabling medical symptoms

Neil Schechter

Boston Children's Hospital, Harvard Medical School, Boston, USA

Physicians are increasingly confronted with patients who present with clusters of vague disabling non-progressive symptoms for which they can offer no concrete medical explanation despite extensive evaluation. These symptoms include abdominal pain, musculoskeletal pain, headaches, fatigue, nausea, mental clouding, and dizziness and have often, as a group, been given many different names with functional, an often misunderstood term, being most frequent. Physicians have responded in various ways to this situation–more and more continued investigation despite a lack of alarm signs, attribution of the symptoms to psychological disorders with referral to mental health providers, or more rarely, by suggesting that the explanation of this cluster of non-specific symptoms is either a controversial disorder (such as chronic Lyme) or more typically, a rare yet previously well-defined entity for which the diagnostic boundaries have been expanded and now accommodate the patient's presentation. Included in this list are mast cell activation disorder, mitochondrial disorders, small fiber neuropathy, Ehlers Danlos syndrome, POTS and others. Although these entities, if appropriately diagnosed, may provide a tangible explanation for the symptoms and offer a specific treatment direction as well as a diagnostic home for the patient, it is also possible that they have triggered or explain only a part of this complex puzzle and do not explain the entire clinical picture which is often more complex, individualized and multifactorial than resulting from a single condition. This talk will review proposed new ICD-11 nomenclature for these entities as well as alternative ways of conceptualizing these complex problems.

## How to start a movement: pain, neuroscience, and exercise

Michael Sangster, PT, Clinical Specialist (Pain Science)

IWK Health Centre, Pediatric Complex Pain Program and Dalhousie University, Halifax, Canada

Conventional physical therapy approaches to the management of complex pain in children are primarily focused on a peripheral structural pathology paradigm in a biomedical context. This limited treatment paradigm fails to consider the recent advances in neuroimaging that have demonstrated structural and morphological cortical changes associated with complex pain presentations. These neuroplastic changes are consistent with the typically observed maladaptive movement patterns in pediatric patients with complex pain and thus have important implications for understanding clinical pain presentations and potential targeted therapeutic interventions. Physical therapists as movement specialists require a fundamental paradigmatic shift to consider a broader understanding of the pain and movement relationship in order to pursue movement intervention in a comprehensive mechanism based neuroscience framework. This presentation will consider the neurobiological analgesia of movement intervention along a movement continuum from novel brain based strategies that harness the movement system without moving to conventional therapeutic movement and exercise. The theoretical frameworks and practical application of emerging novel brain based approaches to the management of pediatric complex pain, including strategies such as pain neurophysiology education to address maladaptive pain cognitions and kinesiophobia, perceptual manipulation to facilitate adaptive motor learning and movement patterns, and exercise as a therapeutic tool to induce neuroplasticity will be discussed.

## Interplay of fear and pain circuitry and impact of rehabilitation

Laura Simons, PhD

Stanford University School of Medicine, Stanford, USA

The fundamental brain circuitry of fear learning has been extensively studied in animals and humans. Brain regions most prominently implicated include the amygdala, hippocampus, and prefrontal cortex, with the amygdala serving as the primary hub of action. As fear learning in the context of pain typically develops after few repetitions, generalizes quickly, and can be maintained simply through anticipation of increased pain, there is tremendous potential to apply the brain circuitry of fear learning to our understanding of individuals suffering with chronic pain. Both clinical and basic research has reported long term consequences of chronic pain in youth. As such the underlying brain circuits that are affected may lie dormant and easily sensitized in subsequent injuries or the altered brain circuits may be the harbingers of chronic co-morbidity (eg, depression). Because the developing adolescent brain is still more “plastic,” effective measures and treatments can rescue individuals from lifelong suffering. Effective rehabilitative treatments for chronic pain involve physical, occupational and cognitive behavioral therapy, reflecting the importance of exposure to previously avoided activities to down regulate fear and amplified pain signaling. In the context of intensive interdisciplinary pain treatment of youth, decreasing pain-related fear is associated with improved physical, psychological, and (cognitive-affective, somatosensory) brain circuit function. A basic understanding on how fear-related processes are entrenched or may be amenable to extinction through eradiation of a fear memory trace via pain rehabilitation can now be evaluated using psychophysiological and brain-imaging techniques.

**Funding:** NICHD/R01083270.

## Barriers to opioid availability for pediatric patients in countries with resource limitation

Sushma Bhatnagar, MD, Anuja Pandit, MD

All India Institute of Medical Sciences, New Delhi, India

No adult is ever prepared to face a child's death. When faced with the news of a child's incurable disease, fear and concern is inevitable for the child, family and health professionals. With recent advances in science, multiple treatment modalities have increased survival, but with increasing complications. Regardless of their age, children and their families suffer from physical, clinical, psychological and ethical aspects of suffering. Pain, often the most prominent symptom, if unaddressed, can have a long-lasting impact on their psyche.

Ideally, pain management for children should follow the same standards as that for adults but children worldwide remain in pain, more so in developing countries. Various studies have categorized barriers to pain relief under 3 groups: patient, system and physician related (1). These barriers revolve around 3 themes: lack of knowledge about holistic pain management, myths and misconception about children's pain and opioid, and limited resources and infrastructure.

“Children do not feel pain,” “Strong opioids should not be used in children”; these common myths continue among physicians.

Restrictions on use of Tramadol (2) and non-availability of strong opioids such as morphine is the foremost cause of unrelieved pain in children. India has amended its draconian narcotic law (3) in 2014 but the lack of knowledge about safe use of opioids for children prevails throughout the country.

Vigorous efforts are needed to incorporate pediatric pain management skills in undergraduate and post graduate medical and nursing curriculum. Protocol-based teaching programs should be developed for pediatricians and oncologists, meanwhile difficult pain should be managed by specialized teams in all the hospitals providing care for children.

We cannot let this indifference linger…!

## Altered interocption and emotional distress in children and adolescents with chronic pain

Tanja Hechler, PhD

University of Trier, Germany

Mounting evidence suggests that interoception, the process of sensing, integrating and interpreting signals originating from inside the body, is altered in adults with chronic pain. Little is known on altered interoception in children and adolescents with chronic pain. Here, we focus on the aspect of affective responding to internal bodily sensations. We hypothesize that via interoceptive fear conditioning, adolescents with chronic pain (aged 11–18 years) will display an erroneous interpretation of internal bodily sensations as a warning sign for impeding pain and will thus show fear responses when confronted with internal bodily sensations. We adapted 2 well-validated research paradigms from anxiety research, the provocation and the imagery paradigm (Gruszka et al. in rev.) to test this assumption. Preliminary findings from our studies reveal that when adolescents are confronted with internal bodily sensations elicited by a standardized muscle tensing task (provocation paradigm) or instructed to imagine bodily sensations typically associated with pain episodes (imagery paradigm), they display fear of elicited internal bodily sensations (self-report, defence response mobilization). Results call for specifically tailored psychological interventions to decrease this fear response such as interoceptive exposure. Interoceptive exposure is considered the treatment of choice to decrease fear of internal bodily sensations. However, very few studies have thus far investigated interoceptive exposure in children and adolescents with chronic pain. It is thus essential to further investigate different forms of interoceptive exposure, such as symptom provocation tasks or imagery-based exposure, regarding their processes, mechanisms and efficacy in children and adolescents with chronic pain.

## The utility of ACT for pediatric chronic pain and the development of a digital intervention

Rikard K. Wicksell, PhD

Karolinska University Hospital, Karolinska Institutet, Stockholm, Sweden

Chronic pain remains to have debilitating effects on a large number of children and adolescents. Medical strategies are normally insufficient, and although studies indicate the utility of interventions based on cognitive behavioural therapy (CBT), improvements are commonly modest and sustained effects are difficult to achieve.

In ACT, as a development within CBT, avoidance of pain-related stimuli is considered central to disability and reduced quality of life. Rather than focusing on alleviation of pain, ACT seeks to minimize the influence of pain on behaviour, ie, *pain interference*. Specifically, the treatment objective in ACT is to improve functioning by increasing the individual's ability to act effectively in accordance with long-term goals and values, ie, behavioral flexibility.

ACT has gained empirical support for adult and pediatric chronic pain. However, the availability of this approach is still very limited.

Despite the empirical support the availability of ACT is still very limited, and a large number of patients do not receive this treatment. In other domains, internet-delivered treatments have been successfully developed to increase accessibility (eg, CBT for anxiety).

Few studies have yet evaluated internet-delivered ACT for chronic pain, and to date there is no empirically supported existing digital intervention based on ACT for pediatric chronic pain.

In this presentation, ACT as a clinical approach will be described. Furthermore, the development of a digital intervention, including pilot data from recent studies with adults as well as youths and parents will be presented

Implications for future research and clinical development will be discussed.

## Children with chronic pain, serious illness, and uncertain prognosis: cases for discussion

Boris Zernikow, MD, PhD^a^, Charles Berde, MD, PhD^b^

^*a*^*German Paediatric Pain Centre Children's and Adolescents' Hospital, Datteln, Germany,* ^*b*^*Boston Children's Hospital, Harvard Medical School, Boston, USA*

Infants, children, and adolescents referred to pediatric pain and palliative care programs cannot be easily dichotomized into those with “chronic non-life-limiting” conditions and those with “advanced illness/end-of-life” conditions. For some of these conditions, longevity is changing due to multiple aspects of supportive care, rather than a single curative therapy. The widespread use of night-time non-invasive mechanical ventilation is an example of a supportive intervention that has contributed to increased longevity for children with chronic respiratory and neuromuscular disorders. Prognosis can also be changed dramatically by novel disease-directed therapies, including antisense oligonucleotides, viral vector-based gene therapies, and stem cell therapies.

Uncertain or changing prognosis has implications for long-term analgesic therapies, for maintaining an orientation towards school and work, and for rehabilitative treatment. We will summarize some of the literature on age-dependence of biological effects of long term administration of analgesics, including NSAIDs, opioids, anticonvulsants, and antidepressants in animals and humans. From electronic record and national database review, we will attempt to summarize some limited information on patterns of long-term opioid prescribing in children.

In this session, we will use a case based approach to discuss managing pain in children with uncertain prognosis by consideration of several cases, including a child with spinal muscular atrophy and a child with epidermolysis bullosa.

